# Linking neuron-glia interactions and longevity

**DOI:** 10.7554/eLife.110158

**Published:** 2026-01-19

**Authors:** Michael Pokrass, Nan Hao

**Affiliations:** 1 Department of Molecular Biology, University of California San Diego La Jolla United States

**Keywords:** aging, glia, cell surface proteome, intercellular signaling, sex-specific regulators, DIP-β, *D. melanogaster*

## Abstract

Proteomics experiments on *Drosophila* reveal sex-specific effects in aging, and an important role for a protein called DIP-β.

**Related research article** Marques MP, Sun B, Park YJ, Jackson T, Lu TC, Qi Y, Harrison E, Wang MC, Venkatachalam K, Pasha OM, Varanasi A, Carey DK, Mani DR, Zirin J, Qadiri M, Hu Y, Perrimon N, Carr SA, Udeshi ND, Luo L, Li J, Li H. 2025. *In situ* glial cell-surface proteomics identifies pro-longevity factors in *Drosophila*. *eLife* **14**:RP109422. doi: 10.7554/eLife.109422.

Aging is often discussed as something that happens inside cells: DNA is damaged, mitochondria stop working, and proteins are misfolded. But aging also changes how cells communicate with each other. For example, neurons in the brain rely on neighboring cells, called glia, for nutrients, waste handling, and local repair. Dysregulation of the interactions between neuron and glia is considered a hallmark of brain aging (Barres, 2008), but the consequences of disrupting neuron-glia communications are still being uncovered.

Many cell-cell communication events occur at the cell surface, where receptors and adhesion proteins let cells sense their environment and connect to their neighbors. However, cell-surface proteins can be difficult to measure for a number of reasons: they are often low in abundance, are often embedded in membranes, and are strongly influenced by the tissue context (Cordwell and Thingholm, 2010). Moreover, standard biochemical approaches can disrupt the contacts between cells that researchers want to understand.

Novel methods for studying cell-surface proteomes in their native context and with cell-type specificity have been recently used to understand neural cell-cell interactions (Li et al., 2020a), but these techniques have not been utilized to understand how cell-cell interactions change in aging brains. Now, in eLife, Hongjie Li (Baylor College of Medicine) and colleagues at Baylor and other institutions across the US – including Madeline Marques as first author – report how they adapted these techniques to study how cell-cell interactions change with age in the fruit fly *Drosophila* (Marques et al., 2025).

The researchers used an adapted *in situ* labeling method to profile proteins at the surface of glia in intact brains taken from the flies. In particular, they compared cell-surface proteomes in young (5 day) and old (50 day) flies to examine how signaling molecules are regulated in the aging brain, and identified a set of 872 proteins that exhibited age-specific differences in abundance. Marques et al. found that proteins that became more abundant with age were enriched for functions related to localization and transport, which supports the idea that older brains may need stronger homeostasis and trafficking control. In contrast, many proteins that became less abundant with age were associated with synapse organization, axon guidance, and related processes. Importantly, their analysis also revealed several proteins that have not been characterized before, thus presenting opportunities to identify new aging biology mechanisms in future studies.

To move from correlation to function, Marques et al. chose 48 genes that exhibited the greatest changes between the glial surface proteomes of young and old flies, and tested whether manipulating these genes in adult glia altered lifespan. These manipulations were made in adult flies because many wiring proteins have essential functions during development, and manipulating them in young flies would cause defects that would obscure their role in aging. Strikingly, this functional screen revealed that many candidate genes have sex-specific effects in lifespan extension, showing results exclusively for male or female animals. This result underscores the notion that aging is not one single program: rather, aging can follow distinct trajectories among individuals within a species (Costa et al., 2025; Jin et al., 2019; Li et al., 2020b). Again, this represents an opportunity for further research, and mechanistic studies of these sex-specific regulators might identify hormonal contexts that tune neuron–glia interactions during aging.

One candidate from the screen, a cell adhesion protein called DIP-β, was found to extend lifespan in both males and females when overexpressed in glia. Older flies with higher levels of DIP-β in their glia also climbed better than controls, suggesting improved late-life function in addition to longer lifespan.

To ask what DIP-β was doing at the molecular level, Marques et al. performed single-nucleus RNA sequencing on whole heads from aged flies, comparing animals in which DIP-β is overexpressed in glia to controls. They also used a computational method called FlyPhoneDB2 (Qadiri et al., 2025) to infer potential changes in cell–cell communications by comparing the expression of certain ligand–receptor pairs across cell types. This analysis suggested that DIP-β overexpression was associated with increased signaling between glia and neurons, and between glia and fat cells, with prominent shifts in a number of signaling pathways (such as the TGF-beta, Wnt, FGFR, and EGFR pathways). This is an appealing model because it connects a surface protein found in glia to broader tissue coordination during aging. It also suggests that DIP-β could act less like a single longevity factor and more like a remodeling factor that changes how glia engage other cell types.

Together, this work provides a cell-type-specific map of how the glial surface proteome shifts with age, and it identifies DIP-β as a surprising adult glial factor that improves late-life phenotypes. The ligand-receptor analysis offers hypotheses about altered intercellular signaling, but key mechanistic questions remain. Does DIP-β act through known binding partners? Does it change glia-neuron contact structure or signaling output? And which subtypes of glial cells are responsible? The strong sex specificity across many hits also points to an under-mapped layer of glial aging biology. Resolving these questions could help extract more general principles of brain aging – and why interventions may work differently in males and females.
